# Diet and Endometriosis: An Umbrella Review

**DOI:** 10.3390/foods14122087

**Published:** 2025-06-13

**Authors:** Lenycia C. L. Neri, Federica Quintiero, Simona Fiorini, Monica Guglielmetti, Ottavia Eleonora Ferraro, Anna Tagliabue, Barbara Gardella, Cinzia Ferraris

**Affiliations:** 1 Laboratory of Food Education and Sport Nutrition, Department of Public Health, Experimental and Forensic Medicine, University of Pavia, 27100 Pavia, Italy; lenyciadecassya.lopesneri@unipv.it (L.C.L.N.); federica.quintiero@unipv.it (F.Q.); simona.fiorini@unipv.it (S.F.); monica.guglielmetti@unipv.it (M.G.); 2 Human Nutrition and Eating Disorder Research Center, Department of Public Health, Experimental and Forensic Medicine, University of Pavia, 27100 Pavia, Italy; anna.tagliabue@unipv.it; 3 ONFoods—Research and Innovation Network on Food and Nutrition Sustainability, Safety and Security—Working ON Foods, 27100 Pavia, Italy; 4 Unit of Biostatistics and Clinical Epidemiology, Department of Public Health, Experimental and Forensic Medicine, University of Pavia, 27100 Pavia, Italy; ottavia.ferraro@unipv.it; 5 Department of Clinical, Surgical, Diagnostic and Paediatric Sciences, University of Pavia, 27100 Pavia, Italy; barbara.gardella@unipv.it; 6 Department of Obstetrics and Gynecology, Fondazione IRCCS Policlinico San Matteo, 27100 Pavia, Italy

**Keywords:** endometriosis, diet, vegetables, caffeine, butter, dairy, umbrella review

## Abstract

The association between nutrition and endometriosis is controversial. This umbrella review aimed to investigate whether specific dietetic strategies are useful for reducing endometriosis risk/symptoms. Systematic reviews on diet therapies for endometriosis were analyzed using the Joanna Briggs Institute (JBI) Manual for Evidence Synthesis methodology, and an umbrella review was implemented using Jamovi software. The 10 included systematic reviews comprised observational studies (cohort, case–control, cross-sectional) and interventional trials (randomized, non-randomized). A mild (class IV, lowest strength on evidence quartile) protective effect on vegetables (RR 0.590; 95% CI 0.49–0.71 *p* < 0.001), cheese (OR 0.840; 95% CI 0.74–0.96 *p* = 0.011), total dairy (RR 0.874; 95% CI 0.81–0.95 *p* = 0.001), and high-fat dairy (RR 0.590; 95% CI 0.81–0.99 *p* = 0.025) was found. Butter (RR 1.266; 95% CI 1.03–1.55 *p* = 0.024) and high caffeine (>300 mg/day) (RR 1.303; 95% CI 1.05–1.62 *p* = 0.019) consumption increased the risk of endometriosis. Other food groups had low-quality evidence due to limited studies. A higher intake of vegetables and dairy products may reduce the risk and/or symptoms of endometriosis, while a high intake of caffeine and butter may increase the risk. However, the heterogeneity across studies is significant, and the overall quality of the findings is low. Therefore, it is crucial to conduct new research in this field, focusing on well-designed randomized trials.

## 1. Introduction

Endometriosis is a debilitating disease characterized by chronic inflammation and the presence of functional endometrial glands and stroma outside the uterine cavity [1]. Endometriosis is a benign disease but it has features similar to malignancies, such as progressive, invasive, and estrogen-dependent growth, recurrence, and a tendency to metastasize [2]. Diagnosis is based on laparoscopy and biopsy for histological confirmation. Treatment can be divided into two forms: pharmaceutical therapies aiming to prevent the growth of the endometriotic implants, and surgical therapies attempting to remove the endometriotic implants [1].

According to data from the Italian Ministry of Health, it is estimated that in Italy, endometriosis affects 10–15% of women of reproductive age and 30–35% of women who are infertile or have difficulty conceiving. There are approximately 3 million women with a confirmed diagnosis, with a worldwide incidence of approximately 176 million new cases. The peak occurs between the ages of 25 and 35, but the disease can also appear in younger age groups [3].

It is a highly diverse disease, exhibiting a wide range of phenotypes and clinical outcomes that can range from no symptoms at all to severe pain and/or infertility, significantly reducing the quality of life [4]. Furthermore, the evidence remains unclear for a complex condition like endometriosis, and symptoms, diagnosis, and treatment remain under discussion [5]. Treatment options include hormonal therapies or laparoscopic surgery to remove the endometriotic lesions [4]; however, they do not exclude the possibility of recurrence. Identifying the factors that contribute to the development and progression of lesions is a key point in achieving new prevention and treatment goals.

The scientific evidence on the association between nutrition and endometriosis, in terms of disease risk, symptom improvement, and quality of life, is rather controversial. Most of the scientific work has evaluated, through observational studies, the potential association between the intake of certain nutrients and foods and the risk of endometriosis diagnosis. A review highlighting the connection between diet and endometriosis emphasized the potential role of anti-inflammatory components in foods in alleviating endometriosis symptoms [6]. Despite this, to date, there are no conclusive data on which nutrients should be monitored when in the presence of a diagnosis of endometriosis.

There are also different systematic reviews trying to synthesize the controversial literature in the field, but no umbrella review has been published on the topic. Brown and Farquhar performed an overview of Cochrane reviews, but the focus was interventions for pain relief and for subfertility in pre-menopausal women clinically diagnosed with endometriosis. No citation of diet or nutrient supplementation was made [7].

The presented umbrella review is focused on exploring which nutritional factors could be related to endometriosis or its symptoms.

## 2. Materials and Methods

This study was divided into two parts. The first part was the systematic review of all published systematic reviews to identify the efficacy and tolerability of the different diet therapies proposed for endometriosis. The systematic review was conducted following the methodology proposed by the JBI Manual for Evidence Synthesis [8]. The second part was the implementation of the umbrella review using the module “Metaumbrella” in Jamovi software (*The jamovi project (2022). jamovi. (Version 2.3) [Computer Software]. Retrieved from https://www.jamovi.org*) [9].

This study was registered in PROSPERO with the number CRD42023432971.

### 2.1. Literature Search

A comprehensive systematic literature search was performed to identify all the published systematic reviews and meta-analyses on the efficacy and tolerability of the different diet therapies proposed for endometriosis. Medline through the PubMed, Scopus, Embase, Web of Science, and Cochrane Library databases, and Google Scholar were searched up to 6 July 2023 without time limitation, and updated on 30 September 2024.

The search strategy included the use of Mesh terms and keywords related to the subject and study design “endometriosis” and “diet” or “nutrient” or “nutrition”. The detailed search strategy for Medline is detailed in the Supplementary Materials (Table S1). The reference lists of selected articles were also manually searched to identify any additional related documents, in addition to consulting relevant publications with experts in the field. The grey literature was not searched, and only published systematic reviews were considered.

### 2.2. Study Selection

This overview only included reviews of dietary components (food groups and/or nutrients) for endometriosis.

The articles that met the following PICOs criteria were included in our study:

(1) Population: Women with endometriosis (defined in original articles as women with or without a laparoscopy/laparotomy test to confirm endometriosis with or without past or concurrent fertility issues).
(2) Intervention/Observation: Any dietetic intervention or observation of dietary components or wider interventions involving dietetic aspects (e.g., interventions approaching physical activity or psychological aspects and diet).
(3) Comparison: A dietetic approach vs. a non-dietetic approach or control.
(4) Outcomes: A reduction in symptoms of endometriosis or risk of endometriosis diagnosis. Due to the unclear definition in published studies, the present umbrella review considered these terms (endometriosis symptoms and risk) as mentioned by the original authors, despite them not being interchangeable.
(5) Studies Included: Systematic reviews and meta-analyses. The inclusion and exclusion criteria are shown in Table 1.

Two authors (LN and FQ) independently screened the titles and abstracts of citations to identify potentially relevant studies. Then, the full texts of potentially eligible articles were obtained and reviewed for further assessment according to the inclusion and exclusion criteria. Controversies were resolved by consulting a third reviewer (CF). Rayyan software (https://new.rayyan.ai/) [10] was used for the study selection process and to remove duplicates.

### 2.3. Data Extraction

Data were extracted from eligible studies by two authors (LN and FQ) independently. A prespecified form in Microsoft Excel was used for this scope. The following information was collected: first author, year of publication, type of review, the systematic review research question, number of articles included, control characteristics, description of interventions/phenomena of interest, search details, sources searched, range (years) of included studies, types of articles included, quality/risk of bias assessment tool reported by the included systematic reviews, outcome assessed, results, significance/direction, heterogeneity assessment, and AMSTAR2 (assessment of multiple systematic reviews) [11] results. Any discrepancy was resolved through discussion with a third author (CF).

#### Risk of Bias Assessment and Quality of Selected Studies

The studies’ risk of bias and quality were assessed independently by the application of 16 questions from the AMSTAR2 instrument by two authors (LN and FQ) (Supplementary Materials—Table S2). Any discrepancy was resolved through discussion to reach a consensus.

All answers “yes” were counted as 1 point, and “no” as 0 points. This way, the final score of risk of bias could reach a maximum of 16 points and a minimum of 0 points, the overall risk of bias being greater in lower scores.

Because the AMSTAR 2 is not supposed to create an overall quality score, the authors decided to consider some criteria for excluding low-quality articles. Articles with a final AMSTAR2 score less than or equal to 5 points (lower tertile) and with 0 points for all the critical questions (2, 4, 7, 13, and 15), considered essential points of the study design, were excluded [11].

### 2.4. Statistical Analysis

We used a random effects model on the data from the papers selected in the first part of this study, using the application in Jamovi software, version 2.5, named “Metaumbrella” [9].

#### Assessment Criteria

The criteria used to classify evidence (Ioannidis’s criteria) were as follows [12]:

1. The heterogeneity among studies using the I2 index;
2. The test to assess an excess of significance;
3. Egger’s regression test to underline small study effects;
4. The largest study significance (where a significant *p*-value (*p* < 0.05) was given to the study with the largest sample);
5. The *p*-value of the pooled effect size (Relative Risk (RR) or Odds Ratio (OR)).

For each item selected, the classification of evidence could vary from class I (strong evidence) to class V (no evidence). The results were also graphed with a forest plot with the pooled effect size and their 95% CI.

## 3. Results

**Figure 1:**
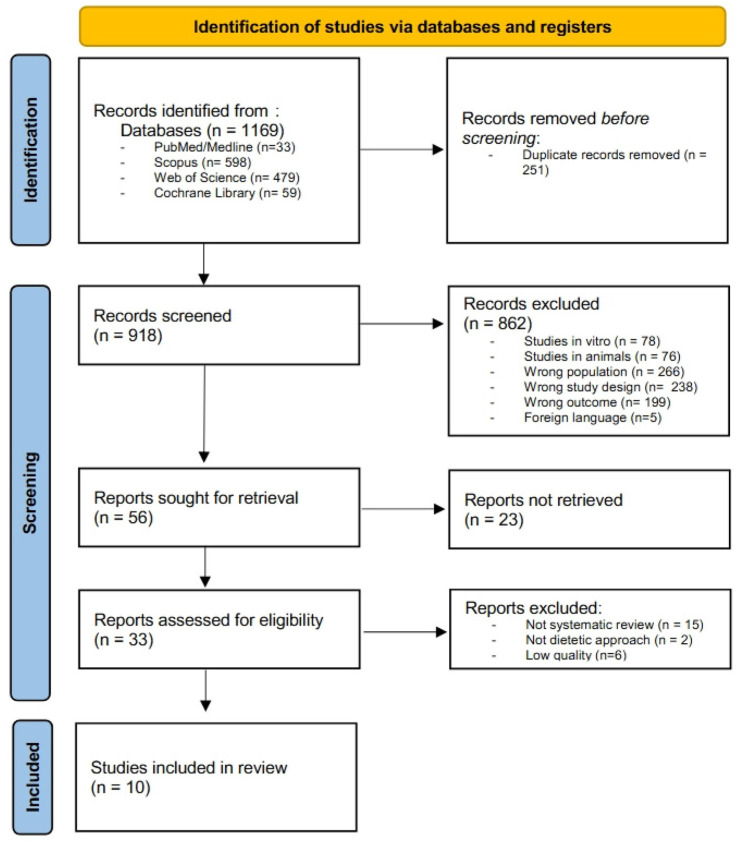
Preferred Reporting Items for Systematic Reviews and Meta-Analyses (PRISMA) flowchart for article selection.

The literature search resulted in 1169 records. After removing duplicated articles (by automatic identification confirmed by the authors), 918 articles remained for the blinded selection made by two authors (LN and FQ). Any conflict was resolved by a third author (CF). Six articles were excluded due to low quality [13,14,15,16,17,18] (their details are reported in Supplementary Materials Table S3). The remaining 10 articles were eligible for inclusion. Figure 1 shows the flowchart of study selection, according to the PRISMA method.

This umbrella review included 10 systematic reviews comprising observational studies (cohort, case–control, cross-sectional) and interventional trials (randomized, non-randomized). These studies were distributed across Europe (n = 4) [19,20,21,22], Asia (n = 4) [23,24,25,26], Oceania (n = 1) [27], and America (n = 1) [28]. The systematic reviews by Nirgianakis et al. [21] analyzed findings from 9 human studies and 12 animal studies. Only data from human studies were considered for this article, and the results were not included in the meta-analysis [21]. It is important to notice that in some cases, the same article was included in more than one of the included systematic reviews, thus resulting in duplication. The statistical analysis was based on five meta-analyses included within these reviews [19,23,24,26,28]. Table 2 provides a detailed description of the selected articles, highlighting the participants’ characteristics, a description of interventions/exposures, the types of articles included, the results, heterogeneity, and the AMSTAR2 score. Details regarding the research questions, databases searched, and quality assessment of the studies are shown in the Supplementary Materials (Table S4). The interventions varied greatly across the included articles: some observational studies focused on food groups and nutrients, while others examined specific food components such as caffeine. The exposures were also heterogeneous, including dietary supplementation and/or specific dietary models like the Mediterranean diet [21]. The results from the risk of bias evaluation through AMSTAR 2 are shown in Table 3. The main concerns that impact the quality of the reviews are presented in Question 7 (list of excluded studies and justification for the exclusions) and Question 10 (sources of funding for the studies included in the review).

**Figure 2:**
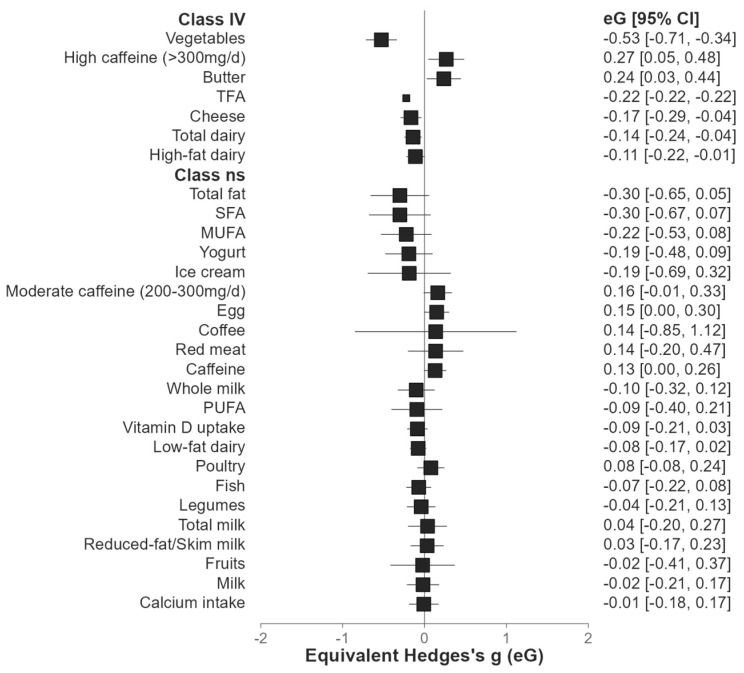
Forest plot from results of meta-analyses included in this umbrella review divided according to the significance in class IV (lower quartile of significance) and class ns (non-significant).

Table 4 presents the crude results from the selected meta-analyses, encompassing studies that focus on nutrients as well as those that examine food products. Figure 2 synthesizes all the results coming from the umbrella review in a forest plot. Five food groups showed weak evidence (class IV, lowest strength on evidence quartile). More precisely, it was found that there was a mild protective effect of vegetables (RR 0.590; 95% CI 0.49–0.71 *p* < 0.001), cheese (OR 0.840; 95% CI 0.74–0.96 *p* = 0.011), total dairy (RR 0.874; 95% CI 0.81–0.95 *p* = 0.001), and high-fat dairy (RR 0.590; 95% CI 0.81–0.99 *p* = 0.025). The nutrients trans fatty acids (TFAs) also seem to show significance in reducing endometriosis risk (OR 0.80), but we found this only in one study. Butter (RR 1.266; 95% CI 1.03–1.55 *p* = 0.024) and high caffeine intake, i.e., >300 mg/day (RR 1.303; 95% CI 1.05–1.62 *p* = 0.019), showed an increased risk of endometriosis. Also, for caffeine (in the case that there was no precisely described amount of caffeine), the model indicated it as a possible risk factor for endometriosis (RR 1.13; 95% CI 1.00–1.28 *p* = 0.044). The evidence for the other food groups, based on Ioannis criteria, was classified as very low-quality (Figure 2) since they appeared in a limited number of studies.

## 4. Discussion

The results of this general review indicate that vegetables and dairy foods may have protective properties against the risk of endometriosis and/or symptom occurrence. On the other hand, butter and high caffeine may increase the risk. Despite the high heterogeneity found by the model in the articles analyzed, the protective effect for endometriosis risk in dairy products is interesting.

Diet is a complex concept affecting health and illness. A healthy diet, primarily focusing on plant-based diets low in animal products, includes vegetables, fruits, legumes, nuts, fish, whole grains, and low-fat dairy, and moderate consumption of red meat, alcoholic beverages, salt, and saturated fatty acids. However, it is unclear if a healthy diet alleviates endometriosis symptoms in women. Although dietary modifications are considered the third most successful self-management measure, no specific diet appears to yield higher self-reported advantages [29].

Dietary patterns, not only single nutrients or food items, have been investigated for their possible impact on endometriosis risk and symptoms [21], particularly those that involve greater consumption of vegetables and dairy products. Although there are conflicting data about symptom improvement, a higher consumption of vegetables and dairy products is generally linked to a lower risk of endometriosis.

Endometriosis is a complex illness, and clinical research on endometriosis and nutrition is equally diverse. To assess the impact of an intervention on a population, it is crucial to base guidelines on well-designed studies with a clear research question, a carefully defined population, and a specific, well-documented intervention. This approach ensures that findings can be generalized to broader patient groups and provides greater clarity on the relationship between diet and endometriosis symptoms [29].

An Italian study [30] revealed evidence of undesirable lifestyle habits in endometriosis patients. In this study, a 6-month Mediterranean diet education intervention (n = 35) improved metabolic and oxidative profiles while significantly enhancing the overall health-related quality of life [30].

Inflammation plays a central role in endometriosis, with immune cells such as cytokines, neutrophils, granulocytes (including mast cells and macrophages), chemokines, and various subsets of T cells driving the inflammatory response. Oxidative stress, a hallmark of chronic inflammation, can be mitigated by antioxidants. Consequently, nutrients with anti-inflammatory properties may help reduce pain symptoms associated with endometriosis [31]. Given the role of inflammation in endometriosis and evidence that reducing dietary fat and increasing dietary fiber can lower circulating estrogen levels, the potential anti-inflammatory effects of plant-based diets have become a topic of clinical interest. Indeed, numerous studies showed strong evidence of the health benefits of consuming a diet high in vegetables and fruits, owing to the presence of bioactive plant compounds such as polyphenols, phytoestrogens, resveratrol, and vitamin C [32].

Lifestyle factors such as poor diet, chronic stress, and unhealthy behaviors can contribute to the development and progression of endometriosis. This condition is characterized by increased levels of reactive oxygen species (ROS) and reduced antioxidant defenses, which lead to cellular damage, inflammation, and the proliferation of ectopic endometrial cells. These imbalances are further aggravated by unhealthy lifestyle habits, which activate immune cells and trigger the release of pro-inflammatory cytokines and mediators. This creates a chronic inflammatory environment in the pelvic cavity, promoting lesion growth, pain, and infertility. Additionally, disease progression is linked to the survival and proliferation of endometrial cells outside the uterus, further worsening the condition. Chronic stress can also negatively affect mental health by dysregulating the hypothalamic–pituitary–adrenal (HPA) axis, increasing systemic inflammation, and contributing to symptoms such as pain, anxiety, and depression in individuals with endometriosis [33,34,35,36,37,38,39,40].

A recent systematic review and meta-analysis [41] of randomized clinical trials on nutritional supplementation was published after the last literature search update and was therefore not included in our umbrella review. Meneghetti et al. [41] reported the results across three categories of interventions: antioxidants (such as resveratrol, and vitamins C, D, and E), anti-inflammatory agents (a combination of nutrients like vitamins, minerals, probiotics, and fish oil), and antiproliferative compounds (such as garlic). The dietary supplementation was found to reduce dysmenorrhea but showed no effect on pelvic pain or dyspareunia. The study highlights that the current body of evidence is characterized by high heterogeneity and a risk of bias.

Despite the complexity of studying food consumption—given the varying intake levels across different populations—the inverse relationship between dairy intake and the risk of endometriosis may be partly explained by the calcium and vitamin D content in dairy products. These nutrients may contribute to the down-regulation of growth-promoting factors, such as insulin-like growth factor-I (IGF-I), and the up-regulation of growth-inhibitory modulators, such as transforming growth factor β (TGF-β) [42]. Dairy, which contains progesterone, estrogen, calcium, vitamin D, and anti-inflammatory components [32], may lower the chance of developing endometriosis by decreasing inflammatory markers such as TNF-a, reactive oxygen species, and interleukin-6 (IL-6). The unfavorable association between vitamin D and C-reactive protein levels has been established in atherosclerotic vascular disease and diabetes mellitus, implying a similar relationship in endometriosis [32,43]. Moreover, regular yogurt consumption, due to its probiotic properties, has been suggested to positively influence the intestinal microbiota [44]. Some studies indicate that probiotics may alleviate symptoms in patients with irritable bowel syndrome (IBS) [45,46,47], a condition that often coexists with or is misdiagnosed as endometriosis [43]. Therefore, it could be plausible that dairy consumption could promote a more beneficial microbiome, potentially reducing endometriosis-related pelvic pain and lowering the risk of visceral hypersensitivity, which might otherwise exacerbate symptoms over time [48]. In addition, there seems to be a positive association also for cheese and high-fat dairy, probably again due to the calcium and vitamin D content.

High-fat dairy products contain not only C12-C16 saturated fatty acids (SFA) but also fatty acids with potential beneficial effects on adiposity and metabolic health. There are studies reporting an inverse association between dairy fat consumption and obesity, metabolic risk factors, and, in some studies, cardiovascular disease (CVD) [49]. Notably, dairy fat is rich in butyric acid (C4:0), conjugated linoleic acid (CLA), cis and trans palmitoleic acid (C16:1), and the branched-chain fatty acid phytanic acid (C20:0). While these fatty acids represent only a small portion of dairy fat, evidence suggests that their modest levels may be physiologically significant, either individually or synergistically [49].

Inexplicably, there also seems to be a positive effect against the risk of endometriosis for trans fatty acids (TFAs), which should be further investigated. In fact, several studies have reported that the consumption of trans fatty acids (TFAs) is associated with an increase in circulating levels of inflammatory markers, including interleukin-6 (IL-6) and tumor necrosis factor-alpha (TNF-α), both of which play a role in the pathogenesis of endometriosis [50,51,52,53].

On the contrary, caffeine consumption has been linked to increased sex hormone-binding globulin (SHBG) concentrations and decreased testosterone levels, raising concerns about its potential negative impact on hormone-dependent conditions like endometriosis [17]. Higher caffeine consumption may be associated with the development of endometriosis; however, there is a lack of well-designed, large-scale clinical trials to explain this link and the role of caffeine in the pathophysiology of endometriosis [32].

The crude results from systematic reviews found that total fat was not significant, but high-fat dairy and butter were. The authors hypothesize that unidentified factors in dairy may protect against endometriosis, but in fat-soluble dairy products like butter, this effect could be reversed. However, a dose–response meta-analysis for butter could not be performed due to the limited number of current studies, making it difficult to propose a hypothesis with an umbrella review.

In an attempt to synthesize the existing literature to establish a consensus on an ideal diet for patients, the challenge lies in reconciling various dietary counseling strategies and approaches. The few available studies exhibit high heterogeneity in several aspects, including the types of food investigated, the populations studied, and the diverse outcomes assessed. This prevents adequate estimation of the necessary indices, making statistical evaluation of the studies impractical (many studies are of insufficient quality).

However, the present umbrella review reinforces the importance of vegetable consumption and suggests some preliminary evidence that may cautiously suggest a re-evaluation of the notion that dairy products promote inflammation. Starting from evidence found in the included works, the findings suggest a possible protective role for certain dairy products, particularly milk and cheese, in the development of endometriosis.

Based on published research, practical and holistic interventions for women with endometriosis may involve adopting lifestyle changes, such as maintaining a physically active routine and following a balanced diet that includes fruits, vegetables, legumes, and dairy products. These changes may help lower systemic inflammation, facilitate detoxification, and relieve uncomfortable symptoms [54,55].

The intricacy of human eating patterns is not adequately captured by the limited focus on just some dietary components (vegetables, dairy, butter, and caffeine). Future research must focus on a wider approach with several food groups and nutrients to perform a valid analysis. Well-designed studies, larger sample sizes, and more homogenous populations are needed to provide more definitive evidence regarding the strength of—or lack of—these relationships. Further exploration is needed about the effectiveness of these recommendations in alleviating endometriosis symptoms within the context of holistic interventions, including nutritional counseling and lifestyle changes, through well-controlled randomized trials.

## 5. Conclusions

This umbrella review suggests that a higher intake of vegetables and dairy products may reduce the risk and/or symptoms of endometriosis, while high intake of caffeine and butter may increase the risk. However, the heterogeneity across studies is significant, and the overall quality of the findings is low. Therefore, it is crucial to conduct new research in this field, focusing on well-designed randomized trials.

## Figures and Tables

**Table 1:** PICOs criteria for study selection.

PICOS Criteria	Inclusion Criteria	Exclusion Criteria
Population	Women with endometriosis	Other pathologies
Intervention	Any dietetic intervention or interventions combined with dietetic aspects or observation of dietary components	Other interventions alone (exercise, surgery, etc.)
Comparison	Dietetic approach	Non-dietetic approach or control
Outcomes	Reduction in intensity (or frequency) of endometriosis (symptoms and risk)	Unrelated to endometriosis
Studies included (types)	Systematic reviews and meta-analyses	Full text not available; no outcomes of interest; randomized controlled trials; uncontrolled observational studies; case reports and case series, opinion articles, guidelines, letters, editorials, comments, news, conference abstracts, theses, and dissertations; and in vitro or animal studies, narrative reviews, scoping reviews, rapid reviews, or mini-reviews
Population	Women with endometriosis	Other pathologies
Research question	Are diet components effective for reducing endometriosis risk/symptoms?	

**Table 2:** Description of the characteristics of the selected articles.

Author, Year, Country	Participants (Characteristics, Total Number)	Description of Interventions/Exposure	Types of Articles Included	Outcome Assessed	Results/Findings	Significance/Direction	Heterogeneity	AMSTAR2
Arab et. al., 2022, Iran [23]	8 studies n = from 156 to 116,607 Age (y) = 18–41.4	Exposure: High dietary intake of selected food groups and nutrients	Case–control, cohort	Endometriosis risk	Total dairy foods: RR 0.90; 95% CI, 0.85 to 0.95; *p* < 0.001, (I2 = 37.0%, *p* = 0.190) Saturated fatty acid: RR 1.06; 95% CI, 1.04 to 1.09; *p* < 0.001, (I2 = 57.3%, *p* = 0.096) Trans fatty acid: RR 1.12; 95% CI, 1.02 to 1.23; *p* = 0.019, (I2 = 73.0%, *p* = 0.025) Red meat: RR 1.17; 95% CI, 1.08 to 1.26; *p* < 0.001, (I2 = 82.4%, *p* = 0.001)	Dietary factors may play a role in the risk of endometriosis ↔	Bias for high-fat dairy (Begg’s test: *p* = 0.117, Egger’s test: *p* = 0.029)	9
Chiaffarino et al., 2014, Italy [19]	8 studies n = 1407 Age (y) = n.r.	Exposure: high versus low or no coffee/caffeine intake	Case–control, cohort	Endometriosis risk	Caffeine: RR 1.26 (95% CI 0.95–1.66) Coffee: RR 1.13 (95% CI 0.46–2.76)	Lack of association	Significant heterogeneity for high coffee/caffeine intake (*χ*2 = 9.37, *p* = 0.053) No evidence of publication bias (*p* = 0.252)	6
Hoorsan et al., 2017, Iran [24]	4 studies n = 2221 cases/70,444 controls Age (y) = n.r.	Exposure: measured by interviews and/or FFQ questionnaires	No restriction	Endometriosis risk	Cheese: OR 0.70 (0.52–0.93), X2: 6.80, *p* = 0.033 and I2: 70.6%. Vegetables: OR 0.43 (0.33–0.57), X2: 1.45, *p* = 0.23 and I2: 30.9%. Fish: OR 0.87 (0.71–1.07), X2:0.86, *p* = 0.65 and I2: 0%.	n.r.	Heterogeneity (Tau2): 0.006, X2: 1.36 (df = 1) and (*p* = 0.243) and I2: 26.6%	6
Huijs et al., 2020, Netherlands [20]	12 studies n = from 1 to 240 Age (y) = n.r.	Intervention: different nutrients, dosages, durations of supplementation (Vitamins B6, A, C, D, E; Ca, Mg, Se, Zn, Fe; lactic ferments; and other nutrients)	Randomized clinical trials, non-randomized clinical trials, retrospective studies, case series, case reports	Endometriosis symptoms	High-quality intervention studies in women with endometriosis are necessary to improve the quality of the evidence.	Nutrients with direct or indirect anti-inflammatory properties are effective in suppressing endometriosis-associated pain	Heterogeneous patient group or a heterogeneous intervention	7
Kechagias et al., 2021, UK, Finland, and Canada [28]	13 studies n = n.r. Age (y) = n.r.	Exposure or intervention: different caffeine-containing beverages (e.g., coffee, tea, cola, chocolate)	Observational studies, case series, randomized controlled trials	Presence of endometriosis	High caffeine consumption (>300 mg/day): RR 1.30, 95% CI 1.04–1.63; I2 = 56%	High caffeine consumption (>300 mg/day): ↑ endometriosis risk	Egger, high heterogeneity between studies	9
Mardon et al., 2022, Australia [27]	15 studies n = 1093; 660 allocated to intervention; 759 to comparator Age (y) = n.r.	Intervention: Dietary supplements (n = 10), dietary modifications (n = 3), over-the-counter (OTC) naproxen sodium (herein referred to as naproxen) (n = 1). Exposure: 3,3’—Diindolylmethane (DIM) supplement; gluten-free diet	Randomized controlled trials, non-randomized controlled trials, cohort studies	Primary outcome: dysmenorrhea, non-menstrual pelvic pain, dyspareunia, overall pain Secondary outcomes: quality of life, side effects, additional medication use	No significant effect	No significant effect	High heterogeneity among studies	11
Nirgianakis K., et al., 2022, Switzerland and Austria [21]	9 studies n = 733 Age (y) = n.r.	Interventions: supplementation of vitamin D; vitamins A, C, and E; omega-3/6; quercetin; vit B3; 5-methyltetrahydrofolate calcium salt, turmeric, and parthenium; Mediterranean diet; low-nickel diet. Exposure: gluten-free diet; low-FODMAP diet	Cohort, case–control, randomized controlled studies	Endometriosis symptoms	Most of the studies reported a positive effect on endometriosis	Dietary interventions: ↓ endometriosis-related pain	High heterogeneity of the interventions and measured outcomes	7
Sukan B., et al., 2022, Turkey [25]	8 studies n = n.r. Age (y) = n.r.	Intervention or exposure: antioxidant supplementation	Randomized controlled trials, observational studies	Chronic pelvic pain, dysmenorrhea, dyspareunia	Use of antioxidant supplementation: ↓ endometriosis-related pain (except 1 study)	Antioxidants could be an alternative treatment method in the management of pain in endometriosis.	High heterogeneity of the studies	10
Sverrisd’ottir U.A., et al., 2022, Denmark [22]	6 studies n = from 31 to 484 Age (y) = n.r.	Intervention: PUFAs, vitamins, minerals, and VSL3 lactic ferments. Exposure: Folic acid metabolite and PUFA supplementation; low-FODMAP diet, a gluten-free diet, and a low-nickel diet.	Randomized controlled trials, observational studies	Pain perception	Diet had a positive impact on pain perception, with all except one study indicating a significant reduction in pain perception	High intake of polyunsaturated fatty acids, a gluten-free diet, and a low-nickel diet may improve painful endometriosis	High heterogeneity of the studies	6
Xiangying Qi, et al., 2021, China [26]	7 studies n = 120,706 Age (y) = n.r.	Exposure: Dairy intake measured by FFQ or structured questionnaire	Case–control, cohort study, randomized controlled trial	Endometriosis risk	High dairy product intake: ↓ endometriosis risk (RR 0.83, 95% CI 0.74–0.93; I2 0%) ↑Total dairy intake (≥21 servings/week): ↓ endometriosis risk; (RR 0.87, 95% CI 0.76–1.00; pnon-linearity = 0.04). Milk consumption (18 servings/week) (RR 0.89, 95% CI 0.80–0.99; non-linearity = 0.022). ↑ high-fat dairy intake: ↓ endometriosis risk compared to low milk intake (RR 0.86, 95% CI 0.75–0.98; I2 0%). Milk consumption ≥18 servings/week (RR 0.86, 95% CI 0.76–0.96; non-linearity = 0.012) ↑ Butter intake: ↑ endometriosis risk compared with low butter intake (RR 1.27, 95% CI 1.03–1.55; I2 0%)	Dairy product intake: ↓ endometriosis risk Dose-dependent relationship, with significant effects (average daily intake ≥ 3 servings)	Inter-study heterogeneity: low to moderate	9

n = number; CI = confidence interval; FODMAP = Fermentable Oligo-, Di-, and Mono-saccharides, And Polyols; FFQ = food-frequency questionnaire; GRADE = Grading of Recommendations Assessment, Development, and Evaluation; JBI = Joanna Briggs Institute; mg = milligrams; MINORS = Methodological Index for Non-Randomized Studies; n.r. = not reported; NIH = National Institute of Health; NOS = Newcastle–Ottawa Scale; OR = Odds Ratio; OTC = over the counter; QUIPS = Quality in Prognostic Studies; PUFAs = polyunsaturated fatty acids; RCTs = randomized controlled trials; RoB2 = Risk of Bias 2.0; ROBINS-I = Risk of Bias of Non-Randomized Studies of Interventions; RR = Relative Risk; STROBE = Strengthening the reporting of observational studies in epidemiology; y = years; I2 and Q Tests = statistical heterogeneity tests.

**Table 3:** Assessment of the methodological quality of systematic reviews according to criteria set by the Center for Evidence-Based Management.

References/AMSTAR2	1	2	3	4	5	6	7	8	9	10	11	12	13	14	15	16	Score
Arab A. et al., 2022 [23]	Y	Y	Y	N	Y	N	N	N	N	N	Y	Y	Y	Y	N	Y	9
Chiaffarino F., et al., 2014 [19]	Y	N	N	N	Y	N	N	Y	N	N	Y	N	Y	Y	N	N	6
Hoorsan H., et al., 2017 [24]	N	N	N	Y	Y	Y	N	N	N	N	N	N	N	Y	Y	Y	6
Huijs E., et al., 2000 [20]	N	N	Y	N	Y	Y	Y	Y	N	N	Y	N	N	N	N	Y	7
Kechagias K.S, et al., 2021 [28]	N	Y	Y	Y	Y	Y	N	Y	N	N	Y	N	N	N	Y	Y	9
Mardon A.K., et al., 2022 [27]	N	Y	Y	Y	Y	Y	N	Y	Y	N	N	Y	Y	N	Y	Y	11
Nirgianakis K., et al., 2022 [21]	N	Y	Y	Y	Y	Y	N	Y	N	N	NA	N	N	N	N	Y	7
Sukan B., et al., 2022 [25]	Y	Y	Y	Y	Y	Y	N	Y	Y	N	NA	NA	NA	NA	Y	Y	10
Sverrisd’ottir U.A., et al., 2022 [22]	N	N	N	N	Y	Y	N	Y	Y	N	NA	NA	NA	NA	Y	Y	6
Xiangying Qi, et al., 2021 [26]	Y	N	N	N	Y	Y	N	Y	N	N	Y	Y	Y	Y	Y	N	9

Y = yes, N = no, NA = not applicable.

**Table 4:** Crude results from selected meta-analysis studies describing the association between endometriosis and food items or diet.

	Arab A. et al., 2022 RR (95% CI) [23]	Chiaffarino F., et al., 2014 RR (95% CI) [19]	Hoorsan H., et al., 2017 OR (95% CI) [24]	Kechagias K.S, et al., 2021 RR (95% CI) [28]	Xiangying Qi, et al., 2021 RR (95% CI) [26]
Total dairy	**0.90 (0.85–0.95)**				0.87 (0.76–1.00)
Milk	0.98 (0.91–1.05)				**0.89 (0.80–0.99)**
High-fat dairy	1.00 (1.00–1.01) *				**0.86 (0.75–0.98)**
Low-fat dairy	0.94 (0.88–1.01) *				
Cheese	0.94 (0.88–1.00)		0.70 (0.52–0.93) *		
Butter					**1.27 (1.03–1.55)**
Total fat	1.00 (0.93–1,08)				
Saturated fatty acid	1.06 (1.04–1.09) *				
Trans fatty acid	1.12 (1.02–1.23) *				
Monounsaturated fatty acid	0.92 (0.82–1.04)				
Polyunsaturated fatty acid	0.93 (0.86–1.02)				
Fruits	0.97 (0.92–1.02) *				
Legumes	1.00 (0.93–1.08)				
Caffeine		1.26 (0.95–1.66)		1.30 (1.04–1.63) **	
Coffee		1.13 (0.46–2.76)			
Vegetables	0.97 (0.92–1.02) *		**0.43 (0.33–0.57)**		
Red meat	1.17 (1.08–1.26) *				
Poultry	1.06 (0.98–1.18)				
Egg	1.06 (0.99–1.15)				
Fish	0.98 (0.91–1.02)		0.87 (0.71–1.07)		

* With evidence of significant heterogeneity. ** >300 mg/day. Highlighted in bold are the risk ratios that indicate a statistically significant effect, whether it is a risk or a protective factor.

## Data Availability

The original contributions presented in the study are included in the article/Supplementary Materials, further inquiries can be directed to the corresponding author.

## Supplementary Materials

The following supporting information can be downloaded at https://www.mdpi.com/article/10.3390/foods14122087/s1: Table S1. Search strategy; Table S2. Questions from the AMSTAR2 instrument—consensus by 2 researchers; Table S3. Details from the excluded articles from the analyses due to poor methodological quality; Table S4. Description of other characteristics of the selected articles.

## Abbreviations

The following abbreviations are used in this manuscript:

AMSTAR2	Assessment of multiple systematic reviews
CI	confidence interval
CLA	conjugated linoleic acid
CVD	cardiovascular disease
FFQ	food-frequency questionnaire
FODMAP	Oligo-, Di-, and Mono-saccharides, And Polyols
GRADE	Grading of Recommendations Assessment Development, and Evaluation
I2 and Q Tests	statistical heterogeneity tests
IBS	irritable bowel syndrome
IGF-I	insulin-like growth factor-I
IL-6	interleukin-6
JBI	Joanna Briggs Institute
mg	Milligrams
MINORS	Methodological Index for Non-Randomized Studies
NIH	National Institute of Health
NOS	Newcastle–Ottawa Scale
OR	Odds Ratio
OTC	over the counter
PICOs	Population; Intervention; Comparison; Outcomes; Studies design
PRISMA	Preferred Reporting Items for Systematic reviews and Meta-Analyses
PUFAs	polyunsaturated fatty acids
RCTs	Randomized controlled trials
RoB2	Risk of Bias 2.0
ROBINS-I=	Risk of Bias of Non-Randomized Studies of Interventions
RR	Relative Risk
SFA	saturated fatty acids
SHBG	sex hormone-binding globulin
STROBE	Strengthening the reporting of observational studies in epidemiology
TFA	trans fatty acids
TGF-β	transforming growth factor β
TNF-α	tumor necrosis factor-alpha
