# Supplementary material for: Diet and Endometriosis: An Umbrella Review
Source: Foods. 2025 Jun 13;14(12):2087. doi: 10.3390/foods14122087 (PMC12192176; doi:10.3390/foods14122087)
Supplement: Supplementary file 1 [file foods-14-02087-s001.zip › foods-3635237-supplementary.pdf]

Diet and endometriosis: un umbrella review.
Lenycia de Cassya Lopes Neri

Table S1. Search strategy.

Database	Search strategy	Results
Pubmed	"Endometriosis/diet therapy"[Mesh]<br>AND<br>(("endometriosis"[MeSH Terms] OR "endometriosis"[All Fields] OR "endometriosis"[All Fields]) AND ("diet"[MeSH Terms] OR "diet"[All Fields]) AND ("therapeutics"[MeSH Terms] OR "therapeutics"[All Fields] OR "treatments"[All Fields] OR "therapy"[MeSH Subheading] OR "therapy"[All Fields] OR "treatment"[All Fields] OR "treatment s"[All Fields])) AND (systematic review[Filter])	33
Scopus	TITLE-ABS-KEY ((“endometriosis” AND (“diet” OR “nutrient” OR “nutrition”))	598
Web of Science	(((ALL=(endometriosis) OR (adenomyosis)) AND (ALL=(nutrition) OR ALL=(diet) OR ALL=(nutrient)))	479
Cochrane	(endometriosis or adenomyosis) and (diet) AND<br>(endometriosis)	12<br><br>47

Diet and endometriosis: un umbrella review.
Lenycia de Cassya Lopes Neri

Table S2: Questions from the AMSTAR2 instrument – consensus by 2 researchers.

Item	Arab A. et al., 2022	Chiaffarino F., et al., 2014	Fjerbæk A., et al., 2007	Hansen S.O., et al., 2013	Heinze N., et al., 2021	Helbig M., et al., 2021	Hipolito-Reis M, et al., 2022	Hoorsan H., et al., 2017	Huijs E., et al., 2020	Kechagias K.S, et al., 2021	Mardon A.K., et al., 2022	Nirgianakis K., et al, 2022	Parazzini F., et al, 2013	Soave I, et al., 2018	Sukan B., et al., 2022	Sverrisd´ottir U.A., et al, 2022	Xiangying Qi, et al, 2021
1. Did the research questions and inclusion criteria for the review include the components of PICO?																	
Population	1	1	0	1	1	1	0	0	1	1	1	0	1		1	1	1
Intervention	1	1	0	1	1	1	0	0	0	1	1	1	1		1	0	1
Comparator group	1	1	0	0	1	0	0	0	0	0	1	0	1		1	0	1
Outcome	1	1	0	0	1	1	0	0	1	1	0	1	1		1	1	1
(Optional) Timeframe for follow-up													1				
Code result: 0 = No, 1 = Yes, NA	1	1	0	0	1	0	0	0	0	0	0	0	1	0	1	0	1
2. Did the report of the review contain an explicit statement that the review methods were established prior to the conduct of the review and did the report justify any significant deviations from the protocol?																	
For Partial Yes, ALL																	
The authors state that they had a written protocol or guide, including (below)	0	0	0	0	0	0	0	0	0	1	1	1	0	0	1	1	0
review question(s)	0	0	0	0	0	1	0	0	0	1	0	1	0		1	0	0
a search strategy	1	1	1	1	1	1	1	1	1	1	1	1	1		1	1	1
inclusion/exclusion criteria	1	1	1	0	1	0	1	1	1	1	1	1	0		1	0	1
a risk of bias assessment	1	1	0	0	0	0	1	1	1	1	1	1	0		1	1	1
For Yes, ALSO ALL																	

the protocol should be registered	1	0	0	0	0	0	0	0	0	1	1	1	0	0	1	1	0
a meta-analysis/synthesis plan, if appropriate, and	1	1	0	0	0	0	0	0	0	1	1	0	0		1	1	1
a plan for investigating causes of heterogeneity	1	1	0	0	0	0	0	1	0	1	1	0	0		1	0	1
justification for any deviations from the protocol	NA	1	0	0	0	0	0	0	1	1	1	0	0		1	0	1
Code result: 0 = No, 1 = Yes, NA	1	0	0	0	0	0	0	0	0	1	1	1	0	0	1	0	0
3. Did the review authors explain their selection of the study designs for inclusion in the review?																	
For Yes, EITHER																	
Explanation for including only RCTs	NA	na	0	0	0	0		0				0					
OR Explanation for including only NRSI	1	0	0	0	0	0		0									
OR Explanation for including both RCTs and NRSI	NA	na	0	0	0	0	1	0	1	1	1	1	0	0	1	0	0
Code result: 0 = No, 1 = Yes, NA	1	0	0	0	0	0	1	0	1	1	1	1	0	0	1	0	0
4. Did the review authors use a comprehensive literature search strategy?																	
For Partial Yes, ALL																	
searched at least 2 databases (relevant to research question)	1	1	0	1	1	0	1	1	1	1	1	1	1	1	1	1	1
provided key word and/or search strategy	1	1	1	1	1	1	1	1	1	1	1	1	1	1	1	1	1
justified publication restrictions (e.g. language)	0	0	0	0	0	0	1	0	1	0	0	1	0	0	1	0	0
For Yes, ALSO ALL																	
searched the reference lists / bibliographies of included studies	1	1	0	1	1	0	0	1	0	1	1	1		1	1	1	1
searched trial/study registries	0	1	0	0	0	0	0	1	0	1	1	1	1	0	1	1	0
included/consulted content experts in the field	0	0	0	1	1	0	0	1	0	1	0	1	0	0	0	0	0
where relevant, searched for grey literature	0	0	0	1	1	0	0	1	0	0	1	1	0	0	1	0	0
conducted search within 24 months of completion of the review	1	1	0	1	1	1	1	1	1	1	1	1	1	1	1	1	1
Code result: 0 = No, 1 = Yes, NA	0	0	0	0	0	0	0	1	0	1	1	1	0	0	1	0	0
5. Did the review authors perform study selection in duplicate?																	
For Yes, EITHER																	
at least two reviewers independently agreed on selection of eligible studies and achieved consensus on which studies to include	1	1	0	0	0	0	1	1	1	1	1	1		1	1	1	1

OR two reviewers selected a sample of eligible studies and achieved good agreement (at least 80 percent), with the remainder selected by one reviewer.	0	0	0	0	0	0	1	1	1	0		1	1				
Code result: 0 = No, 1 = Yes, NA	1	1	0	0	0	0	1	1	1	1	1	1	1	1	1	1	1
6. Did the review authors perform data extraction in duplicate?																	
For Yes, EITHER																	
at least two reviewers achieved consensus on which data to extract from included studies	0	0	0	0	0	0	1	1	1	1	1	1		1	1	1	1
OR two reviewers extracted data from a sample of eligible studies and achieved good agreement (at least 80 percent), with the remainder extracted by one reviewer.	0	0	0	0	0	0	1	1	1	0		1	1				
Code result: 0 = No, 1 = Yes, NA	0	0	0	0	0	0	1	1	1	1	1	1	1	1	1	1	1
7. Did the review authors provide a list of excluded studies and justify the exclusions?																	
For Partial Yes, ALL																	
provided a list of all potentially relevant studies that were read in full-text form but excluded from the review	0	0	0	0	0	0	0	0	0	0	0	0	0	0	0	0	0
For Yes, ALSO ALL																	
Justified the exclusion from the review of each potentially relevant study	0	0	0	0	0	0	0	0	1	0	0	0	0	0		0	0
Code result: 0 = No, 1 = Yes, NA	0	0	0	0	0	0	0	0	1	0	0	0	0	0	0	0	0
8. Did the review authors describe the included studies in adequate detail?																	
For Partial Yes, ALL																	
described populations	1	0	0	1	1	0	1	1	1	1	1	1	1	1	1	1	1
described interventions	1	1	0	1	1	0	1	0	1	1	1	1	1	1	1	1	1
described comparators	0	1	0	1	1	0	1	0	1	1	1	1	1	1	1	1	1
described outcomes	0	1	1	0	1	1	1	1	1	1	1	1	1	1	1	1	1
described research designs	1	1	0	0	0	0	1	1	1	1	1	1	1	1	1	1	1
For Yes, ALSO ALL																	
described population in detail	1	1	0	0	0	0	1	0	1	1	1	1	1	1	1	1	1

described intervention in detail (including doses where relevant)	0	1	0	0	0	0	1	1	1	1	1	1	1	1	1	1	1
described comparator in detail (including doses where relevant)	0	1	0	0	0	0	1	0	1	1	1	1	1	1	1	1	0
described study's setting	0	1	0	1	1	1	1	1	1	1	1	1	1	1	1	1	1
timeframe for follow-up	0	1	0	1	1	1	1	0	1	1	1	1	1	1	1	1	1
Code result: 0 = No, 1 = Yes, NA	0	1	0	0	0	0	1	0	1	1	1	1	1	1	1	1	1
9. Did the review authors use a satisfactory technique for assessing the risk of bias (RoB) in individual studies that were included in the review?																	
For RCTs included in reviews:																	
For Partial Yes, ALL																	
unconcealed allocation, and	0	0	0	0	0	0	1	0	0	0	1	0		0	1	1	0
lack of blinding of patients and assessors when assessing outcomes (unnecessary for objective outcomes such as all-cause mortality)	0	0	0	0	0	0	0	0	0	0	1	0	0	0	1	1	0
For Yes, ALSO ALL																	
allocation sequence that was not truly random, and	0	0	0	0	0	0	1	0	0	0	1	0	0	0	1	1	0
selection of the reported result from among multiple measurements or analyses of a specified outcome	0	0	0	0	0	0	1	0	0	0	1	0	0	0	1	1	0
For NRSI included in reviews:																	
For Partial Yes, ALL																	
from confounding, and	0	1	0	0	0	1	1	0	0	0	1	0	0	0	1	1	0
from selection bias	0	1	0	0	0	1	1	1	1	0	1	0	0	0	1	1	1
For Yes, ALSO ALL																	
methods used to ascertain exposures and outcomes, and	0	1	0	0	0	0	1	0	1	1	1	0	0	0	1	1	0
selection of the reported result from among multiple measurements or analyses of a specified outcome	0	1	0	0	0	0	1	0	1	0	1	0	0	0	1	1	0
Code result: 0 = No, 1 = Yes, NA	0	0	0	0	0	0	1	0	0	0	1	0	0	0	1	1	0
10. Did the review authors report on the sources of funding for the studies included in the review?																	

For Yes, ALL																	
Must have reported on the sources of funding for individual studies included in the review. Note: Reporting that the reviewers looked for this information but it was not reported by study authors also qualifies	0	0	0	0	0	0	0	0	0	0	0	0	0	0	0	0	0
Code result: 0 = No, 1 = Yes, NA	0	0	0	0	0	0	0	0	0	0	0	0	0	0	0	0	0
11. If meta-analysis was performed did the review authors use appropriate methods for statistical combination of results?																	
For RCTs included in reviews:																	
For Yes, ALL																	
The authors justified combining the data in a meta-analysis	1	1	0	na	na	na	0	0	1	1	0	1	1	na	na	na	1
AND they used an appropriate weighted technique to combine study results and adjusted for heterogeneity if present.	1	1	0	na	na	na	0	1	0	1	0	0					1
AND investigated the causes of any heterogeneity	1	1	0	na	na	na	0	1	0	1	0	0					1
For NRSI included in reviews:																	
For Yes, ALL																	
The authors justified combining the data in a meta-analysis	1	1	0	na	na	na		0	1	1	1	0	na			na	1
AND they used an appropriate weighted technique to combine study results, adjusting for heterogeneity if present	1	1	0	na	na	na		0		1	0	0					1
AND they statistically combined effect estimates from NRSI that were adjusted for confounding, rather than combining raw data, or justified combining raw data when adjusted effect estimates were not available	1	1	0	na	na	na		1		1	0	0					1
AND they reported separate summary estimates for RCTs and NRSI separately when both were included in the review	NA	1	0	na	na	na		1		1	0	0					1
Code result: 0 = No, 1 = Yes, NA	1	1	0					0	1	1	0						1

12. If meta-analysis was performed, did the review authors assess the potential impact of RoB in individual studies on the results of the meta-analysis or other evidence synthesis?																	
For Yes, EITHER																	
included only low risk of bias RCTs	na	0	0	0	0	0		0	0	0			na	na	na	na	1
OR, if the pooled estimate was based on RCTs and/or NRSI at variable RoB, the authors performed analyses to investigate possible impact of RoB on summary estimates of effect.	1	0	0		0	0		0	0	0	1	0					
Code result: 0 = No, 1 = Yes, NA	1	0	0	0	0	0		0	0	0	1	0					1
13. Did the review authors account for RoB in individual studies when interpreting/ discussing the results of the review?																	
For Yes, EITHER																	
included only low risk of bias RCTs		0	0	0	0	0		0	0	0			na	na	na	na	1
OR, if RCTs with moderate or high RoB, or NRSI were included the review provided a discussion of the likely impact of RoB on the results	1	1	0	0	0	0		0	0	0	1	0					
Code result: 0 = No, 1 = Yes, NA	1	1	0	0	0	0		0	0	0	1	0					1
14. Did the review authors provide a satisfactory explanation for, and discussion of, any heterogeneity observed in the results of the review?																	
For Yes, EITHER																	
There was no significant heterogeneity in the results		1	0		0	0		1		0				na	na	na	
OR if heterogeneity was present the authors performed an investigation of sources of any heterogeneity in the results and discussed the impact of this on the results of the review	1		0	1	0	0			0	0	0	0					1
Code result: 0 = No, 1 = Yes, NA	1	1	0	1	0	0	0	1	0	0	0	0	0				1
15. If they performed quantitative synthesis did the review authors carry out an adequate investigation of publication bias (small study bias) and discuss its likely impact on the results of the review?																	
For Yes, ALL																	
performed graphical or statistical tests for publication bias and discussed the likelihood and magnitude of impact of publication bias	0	0	0	0	0	0	1	1	0	1	1	0	0	0	1	1	1

Code result: 0 = No, 1 = Yes, NA	0	0	0	0	0	0	1	1	0	1	1	0	0	0	1	1	1
16. Did the review authors report any potential sources of conflict of interest, including any funding they received for conducting the review?																	
For Yes, EITHER																	
The authors reported no competing interests	1	0	0	0	0	1	1	1	1	1		1	1	1	1	1	0
OR The authors described their funding sources and how they managed potential conflicts of interest		0	0	0	0	1	1				1						
Code result: 0 = No, 1 = Yes, NA	1	0	0	0	0	1	1	1	1	1	1	1	1	1	1	1	0
Count of 1 (Yes)	9	6	0	1	1	1	7	6	7	9	11	7	5	4	10	6	9
Count of 0 (No)	7	10	16	15	15	15	9	10	9	7	5	9	11	12	6	10	7
**Sum of Yes out of all non-NA**	9	6	0	1	1	1	7	6	7	9	11	7	5	4	10	6	9

Diet and endometriosis: un umbrella review.
Lenycia de Cassya Lopes Neri

TABLE S3: Details from the excluded articles from the analyses due to poor methodological quality

Author, year, country	Question of systematic review	Participants (characteristics, total number)	Description of interventions/phenomena of interest	Sources searched	Searched up to	Types of articles included	Quality / Risk of Bias Assessment	Outcome assessed	Results / Findings	Significance / direction	Heterogeneity	AMSTAR2
Parazzini F., et al, 2013, Italy	What's the role of diet on endometriosis risk, assessing both nutrients and food groups?	11 studies	Information on diet intake was collected using food frequency questionnaires in seven studies, while in one study the questionnaire focused on caffeine and alcohol intake. Simple 'no' or 'yes' questions were used in one study to evaluate coffee intake	MEDLINE (1966 to 2011), EMBASE (1985 to 2011) and Science Citation Index Expanded (1945 to 2011)	1945 to 2011	Most studies were case–control studies. One cohort study was identified.	The diagnosis of endometriosis has been shown to be more frequent among higher social class and more educated women (Parazzini et al., 1995) that could pay a greater attention to minor health problems, but as well could have a more health-oriented			beneficial effect of some nutrients on endometriosis suggest that further research should be conducted in this area.	pooled estimation was not considered since clinical (study populations), methodological (frequency categorization of exposure) and statistical (adjustment for confounding factors) heterogeneity is present across studies. Potential biases may also be due to the different choice of	5

							attitude toward diet and lifestyle habits.			control groups in the considered studies.	
Fjerbæk et al., 2007, Denmark	What is the role of diet in the pathogenesis of endometriosis and dysmenorrhea?	4 studies<br>(2 animal and 2 case-control)<br>Aggiungere n. of participants and age	Consumption of selected dietary items	Cochrane Library, MEDLINE, EMBASE	June 2006	Case-control Animal	Evidence level - The Scottish Intercollegiate Network	Intake of fruit and green vegetables: risk of endometriosis<br>Ham, beef and other red meat increased the risk	The literature on diet and endometriosis is sparse and<br>inconsistent	n.r.	0

Hansen et al., 2013, Denmark	What is the association between dietary components and endometriosis and/or dysmenorrhoea?	12 studies<br>n = from 45 to 70079<br>Age (y) = n.r.	Methods for measuring dietary intake varied between the studies, and the validity of the method was often not provided	Cochrane Library<br>MEDLINE EMBASE	December 2012	Not specified	Evidence level - The Scottish Intercollegiate Network	not all studies had an exact diagnosis of endometriosis	Fish oils/PUFAs:<br>Vegetables/fruit<br>Red meat/ham<br>No consensus between studies	specific types of dietary fats are associated with endometriosis	n.r.	1
Heinze et al., 2021, Germany	describing the effects of food or diet on the symptoms of endometriosis in women with endometriosis.	20 studies<br>n = from 57 to 116,430	The validity of the results of this review is reduced, in particular, by small sample sizes and retrospective surveys with short evaluation periods	PubMed and Zbmed	from 2000 to 03/2020	in vivo clinical studies	Oxford Centre of Evidence Based Medicine (OCEBM)	not all studies had an exact diagnosis of endometriosis	1-diet rich in n3 fatty acids contributes to the improvement of various endometriosis-associated symptoms<br>2-higher consumption of fats overall<br>was associated with an decreased risk<br>3-reducing the consumption of trans fatty acids and palm fat	diet has an influence on endometriosis-associated symptoms, but no recommendations for any specific endometriosis-specific diet could be derived due to the limited and partly inconclusive nature of the available data.	n.r.	1

Helbig et al., 2021, Germany	1.Can endometriosis be prevented with the right nutrition (preventive approach)?<br>2.Does diet influence manifest endometriosis or the postoperative condition (therapeuticapproach)?	not described	not mentioned	PubMed	from 2000 - 2021	reviews as well as two meta analyses, six case-control studies, two randomised trials and four prospective cohort studies			Alcohol consumption and a diet high in trans fats have been shown to have a negative impact on the occurrence of endometriosis;<br>fruit and vegetables, dairy products,unsaturated fats, red meat, fibre, soy products and coffee are not clear	the data is still insufficient and contentious, few studies are often contradictory	n.r.	1
Soave I, et al., 2018, Italy and Switzerland	What are the potential modifiable risk factors of the endometrisis?	14 studies	self-administered questionnaire:<br>Vitamins A-C-E<br>Vitamin D and dairy products<br>Magnesium	Medline–PubMed, Embase, Lilacs, and Cochrane Library	up to October 2017	Excluded only Review articles, commentaries, and papers on the effect of diet on endometriosis-related symptoms were	none	-	-	Epidemiological data concerning the association between diet and endometriosis are growing but still limited and inconsistent.	high variability of the results is noticeable and the presence of bias must be considered.	4

	Green vegetables and fruits<br><br>Soy and phytoestrogens<br><br>Red meat and butter<br><br>Polyunsaturated fatty acids<br><br>Olive oil and monounsaturated fats<br><br>Trans fats<br><br>Green tea<br><br>Fibers and cereals<br><br>Coffee<br><br>Curcumin	further excluded.	

Table S4: Description of other characteristics of the selected articles**.**

**Author, year, country**	**Question of systematic review**	**Sources searched**	**Searched up to**	**Quality / Risk of Bias Assessment tool reported in selected studies**
Arab et. al., 2022, Iran [23]	Does the consumption of dietary food groups and nutrients affect the risk of endometriosis?	PubMed Scopus<br>Web of Science	September 2022	NOS
Chiaffarino et al., 2014, Italy [19]	Coffee/caffeine influence the risk of endometriosis?	MEDLINE EMBASE	May 2013	n.r.
Hoorsan et al., 2017, Iran [24]	What are the risk/preventive factors related to endometriosis?	ClinicalTrials.gov, PubMed EMBASE<br>Scopus,<br>Google scholar Cochrane ProQuest Iranmedex Magiran SID	From 2000 to 2015	STROBE Statement /Begg's funnel plot
Huijs et al., 2020, Netherlands [20]	Effects of nutrition on symptoms in women with endometriosis	PubMed Cochrane Library	March 2019	GRADE criteria
Kechagias et al., 2021, UK, Finland and Canada [28]	The association between caffeine consumption and endometriosis	PubMed Scopus	n.r.	MINORS
Mardon et al., 2022, Australia [27]	What's the evidence concerning efficacy of self-management strategies for females with endometriosis?	Medline Embase EmCare<br>Web of Science Core Collection<br>Scopus Cochrane Central Register of Controlled Trials	24 March 2021	RoB2, ROBINS-I, GRADE
Nirgianakis K., et al, 2022, Switzerland and Austria [21]	What are the effects of dietary intervention on endometriosis?	MEDLINE COCHRANE	30 April 2020	QUIPS tool

	Sub-questions, including (a) if a certain subcategory of patients are more likely to benefit from a dietary intervention or<br><br>(b) if specific dietary interventions ameliorate certain symptoms while others remain unchanged.			
Sukan B., et al., 2022, Turkey [25]	Does antioxidant supplementation reduce pain associated with chronic pelvic pain, dysmenorrhea, and dyspareunia, in endometriosis patients?	Medline–PubMed Scopus Cochrane Library Google Scholar	Since 2012	JBI for RCT<br><br>NIH (pre-post)
Sverrisd´ottir U.A., et al, 2022, Denmark [22]	What is the association between dietary changes and pain perception in women with endometriosis?	Medline, Embase	1946–20 October 2020	GRADE for RCT<br><br>NOS for observational studies
Xiangying Qi, et al, 2021, China [26]	What is the association between dairy products and the risk of endometriosis?	Pubmed, Embase databases, Cochrane Library, Web of Science	February 7th, 2021	NOS<br><br>Begg's and Egger's test

n = number; CI= confidence interval; FODMAP= Fermentable Oligo-, Di-, Mono-saccharides, And Polyols; FFQ: food-frequency questionnaire; GRADE= Grading of Recommendations Assessment, Development, and Evaluation; JBI= Joanna Briggs Institute; mg= milligrams; MINORS= Methodological Index for Non-Randomized Studies; n.r.= not reported; NIH= National Institute of Health; NOS = Newcastle-Ottawa Scale; OR= Odds Ratio; OTC= over the counter; QUIPS= Quality in Prognostic Studies; PUFAs= Polyunsaturated Fatty Acids; RCTs= Randomized controlled trials; RoB2= Risk of Bias 2.0; ROBINS-I= Risk of Bias of Non-Randomized Studies of Interventions; RR= Relative Risk; STROBE= Strengthening the reporting of observational studies in epidemiology; y = years; I2 and Q Tests= statistical heterogeneity tests.
